# The Role of Physical Activity Prescription in Cardiovascular Disease Prevention Amongst South Asian Canadians

**DOI:** 10.3389/fcvm.2018.00165

**Published:** 2018-11-14

**Authors:** Tharmegan Tharmaratnam, Mina A. Iskandar, Sally Doherty, Katrina A. D'Urzo, Swana Kopalakrishnan, Tyler Cameron Tabobondung, Prasaanthan Gopee-Ramanan, Seyon Sivagurunathan, Nirunthan Sivananthan

**Affiliations:** ^1^School of Medicine, Royal College of Surgeons in Ireland, Dublin, Ireland; ^2^School of Medicine, Royal College of Surgeons in Ireland, Adliya, Bahrain; ^3^School of Kinesiology and Health Studies, Queens University, Kingston, ON, Canada; ^4^School of Medicine, Queen's University, Kingston, ON, Canada; ^5^Department of Family Medicine, Michael G. DeGroote School of Medicine, Brantford General Hospital, McMaster University, Hamilton, ON, Canada; ^6^Department of Diagnostic Radiology, Hamilton Health Sciences Centre, Michael G. DeGroote School of Medicine, McMaster University, Hamilton, ON, Canada; ^7^Department of Family Medicine, Stonechurch Family Health Clinic, Michael G. DeGroote School of Medicine, McMaster University, Hamilton, ON, Canada; ^8^Department of Pharmacology & Toxicology, Faculty of Medicine, University of Toronto, Toronto, ON, Canada

**Keywords:** physical activity, South Asian Canadians, cardiovascular disease, coronary heart disease, preventive medicine, health promotion, behavior change, barriers and facilitators

## Abstract

Unequivocal evidence suggests an increased prevalence of cardiovascular disease (CVD) amongst South Asian Canadians (SACs) compared to other ethnic cohorts, due to a combination of their unique cardiometabolic profile and environmental factors. This unfavorable CVD profile is characterized by an elevated risk of dyslipidemia, high apolipoprotein B/apolipoprotein A1 ratio, hypertension, glucose intolerance, type 2 diabetes mellitus, as well as increased BMI, body fat percentage, abdominal and visceral adiposity. Despite the overwhelming evidence for the effectiveness of physical activity (PA) in circumventing the onset of CVD and in the reduction of CVD risk factors, SACs are among the most physically inactive cohorts in Canada. This relates to a set of common and unique socio-cultural barriers, such as gender, beliefs and perceptions about illness, immigration, unfavorable PA environments, and their high prevalence of debilitating chronic diseases. Several strategies to improve PA participation rates in this high-risk population have been suggested, and include the implementation of culturally sensitive PA interventions, as well as clinician training in PA prescription through workshops that emphasize knowledge translation into clinical practice. Therefore, the purpose of this mini-review is to highlight and discuss: (1) the burden of heart disease in SACs (2) the cardiovascular benefits of PA for SACs; (3) factors affecting PA participation among SACs and how they can be addressed; (4) the impact of culturally sensitive PA prescription on CVD prevention; (5) barriers to culture-specific PA prescription by clinicians, and strategies to improve its use and impact.

## Introduction

**Figure 1:**
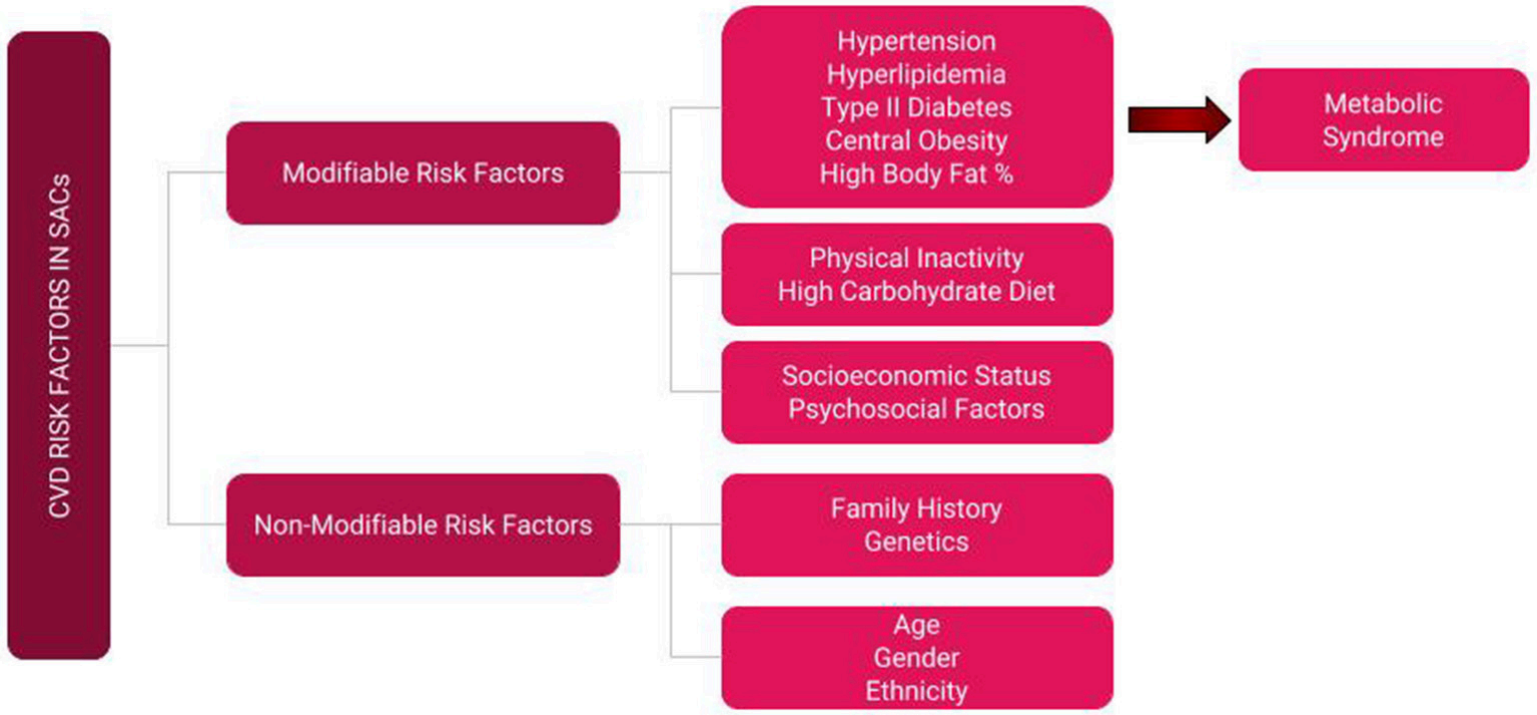
Represents the modifiable and non-modifiable risk factors pertaining to CVD disease.

The term “South Asian Canadian” encompasses diaspora from the sub-Himalayan Asian countries of Bangladesh, Nepal, Pakistan, India, and Sri Lanka. South Asians comprise 5.6% of the Canadian population, and there are presently 1,924,635 individuals of South Asian ethnicity in Canada, accounting for 25.1% of the minority population (1). Considering that South Asian Canadians (SACs) constitute the largest visible minority in Canada, optimizing their health and well-being has important implications for the healthcare system (2). This is particularly important considering the high burden of cardiovascular disease (CVD) in this cohort. Compared to Caucasians and other ethnic groups, SACs have a markedly higher prevalence of cardiovascular disease (CVD) and its associated risk factors (3, 4), which are multifactorial in nature and include genetic predisposition, diet, lifestyle, physical activity (PA) levels, barriers, and attitudes (5). Specifically, SACs have some of the lowest PA rates in Canada (3, 6), attributable to a number of factors to be discussed later. PA behavior is an important modifiable risk factor (Figure 1) for non-communicable disease and presents an important opportunity for the development of targeted interventions to increase PA participation in this high-risk cohort (7). Furthermore, physicians are a respected source of health information and are well-situated to discuss, promote, and prescribe PA as a CVD risk prevention strategy, with previous work demonstrating that clinician-based PA counseling increases patient participation (8–11). In order to make such interventions efficacious, it is crucial to identify the barriers that SACs face in respect to PA, as well as gaps in knowledge clinicians face when prescribing PA to SACs; understanding these challenges may help inform and guide clinicians in successfully formulating ethnoculturally sensitive and compatible health promotion interventions, in an effort to ultimately reduce the high incidence of CVD in this cohort. Therefore, the purpose of this mini-review is to highlight the burden of heart disease in SACs, PA intervention studies in SACs and the reported benefits in this population, factors affecting their PA participation and how to address them, PA prescription as prevention, and gaps in clinician knowledge and possible strategies on how they can be tackled.

## CVD risk factor profile in South Asian Canadians

SACs have one of the highest rates of atherosclerosis and coronary artery disease (CAD) in Canada (12–15). Previous epidemiological work has also shown elevated incidence, prevalence and mortality from CAD in this cohort, which is characterized by two basic features: lower age of onset and elevated severity (14, 16). The INTERHEART study demonstrated that more than 90% of a population's attributable risk to myocardial infarctions (MIs), including that of South Asians, can be explained by 9 key risk factors including smoking, PA, intake of fruits and vegetables, alcohol, hypertension, abdominal adiposity, stress, elevated apolipoprotein B/apolipoprotein A1 (ApoB/ApoA1) ratio, and diabetes mellitus (17). Unfortunately, SACs do in fact suffer from most of these risk factors. Rana et al. demonstrated that, compared to Caucasian Canadians, SACs with similar BMI indices have a higher prevalence of hypertension, as well as higher body fat percentages (3). SACs' profile is also characterized by, increased incidence of type-2 diabetes mellitus, abdominal adiposity, increased serum adipokines, increased physical inactivity, and greater carbohydrate intake (3, 6). They also have alarmingly high rates of glucose intolerance, sub-optimal levels of total cholesterol, triglycerides, LDL particle size, HDL, and other predictive markers (13, 18). SACs do not have higher LDL levels, but they have a larger amount of smaller, denser LDL particles which are more atherogenic than larger LDL particles, which may lead to accelerated atherosclerosis and concomitant multivessel disease (14, 18). In addition to small dense LDL particles and reduced HDL, SACs also have triglyceride (TG)-rich remnants and elevated ApoB and non-HDL-cholesterol (3, 19). TG-rich particles and their remnants are considered highly atherogenic (19, 20), which may exacerbate an already heightened cardiovascular profile.

In addition to CVD, type 2 diabetes mellitus (T2DM) is also a malady amongst the South Asian cohort. Indeed, a population-based repeated cross-sectional study found that, compared to Caucasian, Chinese, and black participants, SAC males had a significant 2.3-fold increase in the prevalence of diabetes over an 11-years period (21). Veenstra concluded that indeed, SACs have the highest rates of diabetes and diabetes-related risk factors among all other ethnic groups in Canada with overarching themes that include greater visceral adiposity indices and lower levels of PA for a given BMI, compared to other ethnicities (22). Another study by Mcqueen and colleagues of 21,465 participants found that South Asians had the highest ApoB/ApoA1 ratio, which has been described as a good indicator for MI risk (23). Taken together, these findings suggest that the co-existence of multiple risk factors provides the optimal milieu for CVD to manifest.

## Physical activity interventions for CVD prevention in SACs

The World Health Organization (WHO) reports physical inactivity as one of the 10 leading causes of death in the Western world, contributing to 1.9 million preventable deaths annually (24, 25). In an effort to combat this substantial mortality rate, the WHO routinely advocates for the promotion of PA to manage chronic disease conditions, such as cardiovascular disease (26). In the PURE Study, Lear et al. examined PA type, amount and their association with mortality in 130,000 participants across 17 countries, including Canada (27). Moderate (150–750 min/week) and high (>750 min/week) PA were associated with a statistically signification reduction in CVD and all-cause mortality. This has important implications for high-risk populations, such as SACs. Indeed, a number of studies conducted in Canada showed the positive impact PA has on the SACs population in reducing CVD risk factors (Table 1) and their predictors. For example, Lesser et al. showed that a 12-weeks PA intervention significantly reduced total abdominal adipose tissue, visceral adipose tissue (VAT), subcutaneous abdominal adipose tissue, waist circumference (WC), and body fat percentage amongst SACs (29, 30). They also found an overall improvement in aerobic fitness, and an association between changes in VAT and changes in glucose and insulin sensitivity. Additionally, Vahabi and Damba observed that after a short 6-weeks PA intervention aimed at SACs, their BMI and WC had decreased, albeit non-significantly (33). The benefits of PA are also comparable to that of pharmacological therapies for certain conditions, such as stroke and secondary management of CVD, yet it is underprescribed (7, 34). Additionally, despite the elevated CVD risk of SACs and studies demonstrating improvements in CVD risk factors with PA in this population, few interventions have been created, tested, and implemented in clinical practice (11).

**Table 1:** A summary of previously published studies examining the role of physical activity in South Asian Canadians.

**Study Name**	**Purpose**	***N* and physical activity intervention**	**Methods**	**Key findings**	**Conclusion**
A pilot examination of a Mosque-based physical activity intervention for South Asian Muslim women in Ontario, Canada [Banerjee et al. 28]	To identify the feasibility and effectiveness of a mosque-based PA intervention for South Asian Muslim women in Canada.	*N* = 62 South Asian Muslim women allocated to a 24-weeks Mosque-based PA intervention.	The feasibility, effectiveness, and the acceptability of the program were assessed using questionnaires (Duke Activity Status Index [DASI] and International Physical Activity Questionnaires).	•Pre-and post-intervention analysis using questionnaires demonstrated increased (^*^) scores of self-efficacy. •Improved (^*^) understanding of PA participation. •Improvement in DASI scores, indicative of improved aerobic capacity and quality of life	Providing culturally-sensitive PA interventions for South Asian Muslim women is effective in improving their fitness and understanding of the importance of PA.
Association between exercise-induced changes in body composition and changes in cardiometabolic risk factors in post-menopausal South Asian women [Lesser et al. 29]	Assess the correlation between changes in total abdominal adipose tissue (TAAT), visceral adipose tissue (VAT), subcutaneous abdominal adipose tissue (SAAT) [TAAT–VAT], waist circumference (WC), % body fat, and BMI in relation to cardiometabolic risk factors after a 12-weeks physical activity (PA) programme.	*N* = 49 sedentary post-menopausal South Asian women randomly allocated to aerobic PA, cultural Bhangra^1^ dance, or control for 12 weeks.	Pre- and post-intervention assessments •CT scan for TAAT, VAT, and SAAT. •Body composition analysis via dual-energy x-ray absorptiometry (DXA), •Blood samples for various cardiometabolic markers (such as glucose, HDL and LDL), •PA testing using the Bruce Protocol.	•Reduction (^*^) in VAT, TAAT, SAAT, WC, % body fat, and glucose post-intervention. •Association (^*^) between changes in VAT and changes in glucose and insulin sensitivity. •Improvement in aerobic fitness (VO_2peak_).	Inactive South Asian women should partake in aerobic PA to reduce the development of cardiometabolic pathologies and to improve their cardiometabolic risk profile.
Effectiveness of exercise on visceral adipose tissue in older South Asian Women [Lesser et al. 30]	To determine whether PA can reduce VAT in SACs.	*N* = 75 sedentary post-menopausal South Asian women randomly allocated to standard gym-based program, cultural Bhangra^1^ dance, or control for 12 weeks.	Pre- and post-intervention assessments •CT scan for TAAT, VAT, and SAAT. •Body composition analysis via DXA. •Blood samples for glucose and insulin levels. •PA testing using the Bruce Protocol.	•Attendance was greatest for the Bhangra^1^ group compared to the standard gym-based PA group. •Reductions (^*^) in VAT compared to control.	Considering the higher adherence in the cultural Bhangra^1^ group, tailored and cultural PA programs may be important to improve PA compliance.
Gender-associated perceptions of barriers and motivators to physical activity participation in South Asian Punjabis living in Western Canada [Caperchione et al. 31]	To elucidate the gender-associated facilitators and barriers to PA participation in SACs residing in Western Canada.	N = 240 South Asian Punjabi adults. No intervention.	Computer-assisted telephone interviews pertaining to their perceptions of facilitators and barriers to PA.	•Men reported disease prevention and reduction of disease as motivators, and the climate as a barrier. •Women reported reducing their weight as a motivator, and a lack of time and motivation as barriers.	Gender-associated differences exist in regard to PA participation.
The association between cardiorespiratory fitness and abdominal adiposity in postmenopausal, physically inactive South Asian women [Lesser et al. 32]	To assess cardiorespiratory fitness (CRF) [using VO_2peak_] and its correlation with body fat distribution and abdominal adiposity in post-menopausal SAC women.	*N* = 55 sedentary post-menopausal South Asian women from Vancouver, Canada. No intervention.	Cross-sectional analysis of a registered RCT •VO_2peak_ measured with Bruce Protocol. •TAAT, VAT, and SAT measured with CT scan.	Compared to women in lowest third of VO_2peak_, those with the highest VO_2peak_ had lower (^*^) abdominal adiposity, visceral fat, and subcutaneous abdominal adipose.	•VO_2peak_ values are negatively correlated to adiposity in post-menopausal SAC women, independent of age and adiposity levels.
			•DXA used to assess body composition.		•Improvements in cardiorespiratory fitness via improvements in VO_2peak_ may thus favorably alter the physiological profile in this high-risk group.
The association between physical activity and liver fat after five years of follow-up in primary intervention multi-ethnic cohort [Lesser et al. 32]	To identify if amount and intensity of PA at baseline are correlated to liver fat (LF).	*N* = 478 healthy men and women of Aboriginal, Chinese, European, and South Asian backgrounds living in Vancouver, Canada. Baseline PA measured in 2004–2005.	Baseline measurements •Questionnaire on PA to quantify level of PA. •Demographic data. •BMI and WC. •Total and % body fat using DXA. 5-years follow up •CT scan to measure LF. •Blood samples for cardiometabolic markers. •PA questionnaire. •Anthropometric data.	Vigorous PA and moderate to vigorous PA (MVPA), but not moderate PA, were good predictive indicators of LF at 5 years follow-up.	Vigorous PA is important to prevent LF accumulation and prevent the onset of associated co-morbidities.
A feasibility study of a culturally and gender-specific dance to promote physical activity for South Asian immigrant women in the Greater Toronto Area [Vahabi and Damba, 33]	To better understand the health impacts and feasibility of implementing a cultural and gender-specific PA intervention among South Asian women in Toronto.	*N* = 27 minimally physically active South Asian women from the Greater Toronto Area, Canada exposed to a 6-weeks Bollywood Dance PA program (2 × /week), led by a South Asian instructor.	•Demographic data taken. •Baseline PA level measured using the Godin Leisure-Time Exercise Questionnaire. •Pre- and post-intervention interviews regarding physical/social/mental health, satisfaction, barriers, and enablers. •Pre- and post-intervention anthropometric data taken. •Participants' adherence taken intra-intervention.	BMI and WC decreased post-intervention. Participants were satisfied with the dance program and reported physical health benefits such as increased energy, self-esteem, and feeling more encouraged to have a more active lifestyle.	Bollywood dance is a culturally sensitive and feasible intervention to engage PA participation amongst South Asian women in Canada.

*To provide local contextualization, only studies conducted in Canada were included*.

1 *A traditional Indian folk dance*.

(^*^) Denotes significance.

## Barriers to physical activity in SACs

Despite the established benefits of PA as a CVD prevention and management strategy, and a well-established modifiable risk factor for non-communicable diseases, participation rates are lowest in SACs, compared to other ethnic groups (3, 6). A cross-sectional study of 171,513 participants concluded that SACs are the least physically active ethnic group in Canada (35). The low participation rates in PA in SACs are related to the barriers highlighted below.

## Culture and illness perception

South Asian cultures are diverse yet unique, with superseding concepts of cultural suitability and fatalism (36, 37), in addition to overarching holistic and spiritual views of disease and illness (38–43). Illness is often attributed to fate, with limited perceived jurisdiction over the matter (44–46). A study surveying South Asian and Caucasian patients with CVD in Canada demonstrated that SACs patients were less likely to perceive that they had control over their illness and that their illness was unpredictable, cyclical in nature, and a result of fate (46). Therefore, such beliefs may partly explain the low PA participation levels (36). Additionally, previous work has shown that South Asians' beliefs and attitudes have a significant effect on health outcomes, as health beliefs influence health behavior, such as a lower locus of control being associated with an increased risk of MI occurrence (47–49). To combat this issue, modifying illness perception is important to ensure that these individuals believe they have capacity to improve their cardiovascular health (50). By altering illness perception through education, SACs may be more inclined to participate in PA as a form of primary and secondary prevention (46).

Overweightness is often considered a desirable trait in South Asian cultures–especially in children–as they represent affluence and health (37, 51–53). Indeed, a cross-sectional study by Banerjee et al. demonstrated that SAC children (particularly boys) aged 10–12 have a greater prevalence of being overweight, compared to non-SACs (54). A more in-depth discussion on barriers faced specifically by SAC children and adolescents can be found under the “Barriers in South Asian Children” section below.

## Immigration

Immigration is a life-changing process that brings with it the necessity to acculturate into Western society, climate, diet, and way of life (55). Initially, new South Asian immigrants may find themselves struggling with PA participation due to their aforementioned native cultures and traditions, but indeed PA levels increase with increasing time since immigration (56, 57). Those born outside of Canada are also more likely to be physically inactive than Canadian born counter-parts and established immigrants (58), highlighting the importance of acculturation in increasing PA levels (57). Based on the Canadian Community Health Survey, Mahmood and colleagues reported that 60% of recent immigrants were inactive compared to 63% of established immigrants, with those from visible minorities, such as South Asians (59). Although PA participation has been shown to increase with length of residency, Chiu and colleagues reported SACs CVD risk factor profile worsens with increasing residency duration in Canada, with no significant difference in CVD prevalence. Elevated risk factor profile with increasing residency duration may be attributed to factors, such as worsening dietary habits and lifestyle associated with increased exposure to Western diets and urbanization (14, 60). Creatore and colleagues also reported that longer term Ontario residents had a significantly higher prevalence of diabetes, compared to recent immigrants (61). This disparity may be partly explained by the thrifty gene hypothesis, where individuals conditioned to a nutritious diet with low-fat/low-salt, high nutrient and PA levels succumb to the high-fat/high-salt diets with concomitant reductions in PA levels in Western society. As a result, immigrants from South Asian countries are particularly susceptible to the shift in lifestyle that accompanies immigration (62, 63), and this has been shown to result in increased mortality from CAD and MI, as well as reduced health-related quality of life after treatment for MI (64, 65). Indeed, the effect of immigration on immigrants' cardiometabolic profile is not only isolated to SACs; numerous ethnic groups have showcased a similar decline in their cardiometabolic health and increase in risk factors. A well-documented example pertains to Japanese immigrants to the United States (US). In numerous seminal studies comparing and contrasting a Japanese cohort living in the States to a Japanese cohort in Japan, the former group was not only found to have a higher BMI, but also a lower a glucose tolerance than the latter group (66–70). Additionally, Curb et al. reported that those living in the US had twice as much fat in their diet than those living in Japan (71).

## Gender differences

South Asian culture typically emphasizes physical separation between men and women (72), which dissuades South Asian women from participating in non-gender specific PA programs (73). A systematic review examining themes regarding PA among South Asian women identified mixed-sex facilities and male instructors as a common barrier (74). Furthermore, Caperchione et al. identified other barriers to PA in SAC women living in Western Canada, such as insufficient time due to family and work commitment for PA (31). Indeed, SAC women participate in PA less than their male counterparts (75), and are 2.83 times more likely to be physically inactive than white Canadians (57). In order to combat low participation rates, implementing community-based PA programs in a culturally-sensitive and gender specific manner may improve adherence and participation levels. Numerous studies support this finding (28–31, 33) (Table 1). The low PA rates in SAC women may explain why many of the PA interventions studies have focused on this cohort, as well as on high risk sub-groups, such as post-menopausal women (29, 30, 32), whom have a heightened risk of cardiovascular disease, presumably due to a decrease in the cardioprotective effects of estrogen (76). To combat the low participation rates seen in SAC females, public PA venues may incorporate female-only days or sessions within their schedule. Another possibility is to encourage local culture centers, such as mosques, to incorporate female-only cultural PA interventions, many of which had been piloted and deemed successful (28–30, 33).

## Physical activity environment

PA environments are a subdivision of the physical environment around us, and may be natural or built. Built environments include any space, such as homes, schools, and gymnasiums, as well as objects, such as treadmills and bicycles. Natural environments include any unaltered spaces or objects, such as open green fields or trees, respectively. Such spaces or objects and their policies then influence PA (77). It is well-known that PA environments differ between races (78). For example, Khan et al. showed that SACs often have less home exercise equipment and fewer convenient PA venues (79). SAC men have also previously reported the weather as a barrier (31, 80). Another reported issue among SAC adolescents is the lack of safe PA environments (81). To combat SACs unfamiliarity with their PA environment and concerns regarding safety, physicians and allied health professionals should be equipped with knowledge about PA facilities, programs, and resources within the community, and reassure parents and adolescents alike regarding their safety concerns.

## Chronic disease

An often-understated point in the hindrance of SACs PA participation is their immensely high rate of chronic disease; SACs rates of chronic diseases, such as hypertension, diabetes, atherosclerosis, and coronary artery disease are among the highest in Canada (3, 6, 12, 13, 15, 21, 22). Such a high prevalence of chronic disease may explain their low rates of PA. Indeed, it is well-known that chronic disease negatively affects PA levels since, among many other reasons, affected patients often have less energy to expend on PA (82). However, in addition to the actual burden of the disease, SACs may suffer from additional barriers not directly related to disease. For example, a study exploring PA among Sikh Canadians with previous MIs had not only identified fatigue and weakness, but also migration-related issues and a lack of knowledge regarding safe limits of exertion as major barriers to PA (83). The problem here is 2-fold. Firstly, there is a lack of knowledge regarding the importance of PA in reducing onset and effects of chronic disease, as well as a lack of knowledge regarding levels of safe PA in those with chronic disease. Secondly, a causality dilemma manifests: which came first, the disease or the inactivity? As such, clinicians should incorporate the importance of PA in their discussion with patients. They should be equipped with information regarding safe exertion levels for those with highly disabling conditions like CVD, and educate patients about the importance of PA for harm reduction in regard to the onset of CVD associated risk factors. Despite this, cardiac rehabilitation referral by physicians remains low in SACs, Therefore, endorsing and referring patients to PA programs is a possible strategy that may lead to improved survival and accrued benefits amongst SACs (84).

## Physical activity in South Asian Canadian children

Childhood is a critical period for adopting healthy lifestyles and behaviors. Obesity during childhood and adolescence has been associated with the development of hypertension, diabetes, and cardiovascular disease in adulthood (85). SAC adolescents have worse cardiovascular risk factors (lower HDL levels and higher triglyceride levels) when compared to adolescents of European or Chinese descent (86). Interestingly, dietary intake does not significantly differ amongst the ethnic groups, which suggests that other factors, such as obesity and low levels of physical activity are contributing to the higher rates of dyslipidemia seen in SAC adolescents (86). However, more recently, consumption levels of sugar-sweetened beverages, such as those sweetened with fructose, has been associated with elevated BMI amongst children living in British Columbia, suggesting that this may be one contributing factor to the elevated obesity rates observed in this cohort (87). Furthermore, this may predispose SAC children to develop other co-morbidities in addition to metabolic syndrome, such as non-alcoholic fatty liver disease (NAFLD). Presently, NAFLD is an emerging epidemic amongst the South Asian population and the Western world, and is characterized by macrovesicular fatty infiltration of at least 5% of liver tissue (88, 89). PA implementation and encouraging sports participation at a young age, are all effective preventive measures which may protect SAC children from developing non-communicable disease in later life.

## Barriers in South Asian children

SAC adolescents identify several barriers impeding PA participation, some of which overlap with barriers that SAC adults experience. SAC adolescents report feeling limited to PA participation due to a lack of outdoor space, not living in close proximation to a gym or school, and due to parental concerns about safety (81). Furthermore, South Asian culture heavily emphasizes academic achievement and performance (90). As such, SAC students feel the need to prioritize studying time in order to achieve academic success (81). SAC girls report unique barriers to PA participation including concerns about physical appearance and feeling a lack of intrinsic motivation to be physically active due to societal norms that undervalue women's performance in sports (81). Encouraging PA participation during childhood years can reduce the prevalence of obesity and cardiovascular risk factors. PA programs and health promotion tactics geared toward children should consider ways to encourage female PA participation, identify methods to improve physical activity in urban areas lacking outdoor space, and how to incorporate physical activity effectively into students' lives without increasing academic pressure (81).

## Physical activity amongst South Asians in other Western Nations

Outside of Canada, the US and United Kingdom (UK) have two of the largest South Asian diaspora populations in the world. And indeed, the aforementioned unfavorable trends in metabolic profile and PA behavior hold true. For example, in the US, studies have shown that South Asians suffer from higher plasma LDL, triglycerides, fasting insulin levels (91), as well as fasting glucose levels (92, 93), compared to their Caucasian counterparts. Additionally, South Asians in the US have been shown to develop early-onset excess body fat, dyslipidemia, T2DM, and atherosclerosis, irrespective of their geographical location (94). Moreover, numerous studies have shown that South Asian in the US are less likely to participate in PA and meet the minimum PA guidelines, compared to Caucasian Americans (75, 95, 96). In one study, only 52% of participants were reported to meet the recommended daily PA guidelines as measured by accelerometers. This may be attributed to a lack of understanding and awareness about how exercise can modulate the cardiometabolic risk profile. Indeed, Bangladeshi immigrants in the US, were shown to have a lower understanding about CVD risk factors compared to the general Caucasian population, attributing diet and cholesterol as risk factors. They were also less likely to mention sedentary behavior as a CVD risk factor. To ameliorate this, the American Heart Association, recommends that tailored interventions that incorporate the cultural context are the best approach to improve success of both exercise and dietary interventions in South Asians (97).

South Asians in the UK portray congruent metabolic profiles and PA behaviors. A cross-sectional study conducted by Hayes at al. showed that, in comparison to the European population, South Asians participated less in PA, had higher BMI indices, systolic blood pressure measurements, and blood glucose levels (98). In fact, Fischbacher et al. concluded that all South Asian cohorts are the among the least physically active groups in the UK (99). Moreover, an 11-years follow-up cohort study by Mather et al. reported that British South Asians were 4 times more likely to report MIs than Caucasians, and that almost 80% of deaths of South Asians were due to circulatory disease, compared to 46% of Caucasians (100). As such, it is clear that South Asians' unfavorable cardiometabolic profile and low PA participation is an issue not isolated to only those living in Canada. It also appears that inter-generational differences affect PA levels, as second-generation South Asians are more active than first-generation immigrants, but still less active compared to the general Caucasian British population (101). Iliodromiti and colleagues suggest that South Asian men and women in the UK should undertake 230 min/week, as opposed to the standard 150 min/week in order to obtain the same cardiometabolic risk factor profile score, as Caucasians (102). However, to the best of our knowledge, no randomized-controlled trial (RCT) to identify if increased PA time/week can moderate cardiometabolic risk in this cohort has been conducted nor validated. Considering that South Asians are already amongst the least active ethnic cohorts in the UK, studies advocate for tailoring interventions that incorporate the social context of people's lives, and are culturally appropriate (103).

## Addressing physical activity participation barriers

The Canadian Society for Exercise Physiology (CSEP) has developed age-specific PA guidelines in an effort to promote healthy living and prevention of disease (104). However, health promotion tactics, such as the ones implored by CSEP are developed for the general population. In order to address barriers to PA participation in a specific population, such as SACs, a holistic approach needs to be undertaken: one that encompasses gender, cultural, and social differences, among others mentioned earlier. In Canada, Aboriginals and SACs are the two major populations that experience elevated rates of chronic diseases and the highest rates of CVD (105). Previous work has shown that culturally responsive interventions that implore gender-sensitive and culturally specific approaches are effective in heart health promotion among indigenous women (106). Following the intervention, indigenous women became more mindful of the importance of PA in improving health outcomes. PA programs in religious institutions, such as mosques have also been effective in promoting PA in minority groups (28, 107). By integrating PA programs into religious institutions, minority groups are motivated to participate as this provides them with a safe and familiar environment (107). Women identified several facilitators for engagement in such programs including personal health benefits, social support networks, an environment which respects both cultural and religious views (107), suggesting the importance of culturally-sensitive approaches in PA programming to ensure high-uptake. Likewise, previous interventions aimed at SACs were more effective when adapted to their socio-cultural context (28, 30, 33, 108–110). For example, Lesser et al. (30) found that post-menopausal SAC women adhered more to a culture-based PA intervention than a control gym-based intervention. Vahabi and Damba showed that a 6-weeks culture-based PA intervention for SAC women had high rates of adherence, overall satisfaction, and positive comments, such as increased energy, self-esteem, and feeling more encouraged to have an active lifestyle; the only negative criticism from participants was that the intervention was too short (33). Similarly, Banerjee et al. noted that, after a mosque-based intervention, participants had self-reported increased self-efficacy and an improved understanding of PA participation (28). Additionally, it is of importance to note that not all patients will have the same barriers and facilitators to PA uptake. Thus, the use of an evidence-based behavior change framework, such as the Theoretical Domains Framework (TDF) may be valuable in identifying the key barriers that pertain specifically to the individual patients at hand, and ultimately, in designing more suitable and specific interventions for them (111, 112). Certainly, evidence-based interventions have been shown to be effective in lowering risk factors and mortality in high-risk populations, such as SACs (11), and the TDF has been previously successful in informing such interventions to increase patients' PA participation (113, 114).

## Clinician knowledge in physical activity prescription

There is a significant body of evidence supporting the potency of physician counseling and advice in increasing patients' PA levels (8–10). However, a recent study by O'Brien et al. reported that Canadian physicians have insufficient knowledge and experience discussing PA with their patients (115). Only 9% of physicians self-reported being exceptionally knowledgeable in this area. However, steps are being made to address this. In 2012, CSEP, adopted the Exercise is Medicine Canada (EIMC) initiative, 5 years after its implementation in the US by the American College of Sports Medicine (116). As a nation-wide strategy, EIMC aims to implement chronic disease prevention strategies to reduce sedentary behavior and physical inactivity, to foster collaboration with clinicians and allied health professionals (AHPs) to combat and prevent chronic disease, and to increase the proportion of clinicians and AHPs who incorporate PA prescription into their practice (117). Indeed, EIMC and their on-campus chapters have implemented nation-wide workshops that educate medical students, physicians and AHPs about the importance of PA prescription (115). A recent study by Fowles et al. examined physicians across 7 provinces in Canada, and their practices and perception regarding PA prescription following an EIMC workshop (118). Physicians completed questionnaires at baseline and 3 months' post-workshop. Three months after the EIMC workshop, physicians were significantly more confident compared to pre-workshop, with barriers, such as lack of time and resources seen as less of an impediment, leading to an overall increase in physicians providing PA prescriptions for patients. Another study by Brennan et al. (119) assessing medical student social cognitions toward exercise counseling demonstrated improvements in attitudes, perceived behavioral control and intentions to discuss PA with future patients from pre- and post-workshop. Additionally, Windt et al. demonstrated that a 3-h education workshop significantly increased the percentage of physicians who provided written PA prescription, 4 weeks post-workshop (120). The Khush Dil (Happy Heart) Study demonstrated that health visitor-led screening clinics and workshops offering nutrition education, cooking workshops, aerobic exercise for women and circuit training for men resulted in increased motivational status, improved physical activity levels, and reduced risk factor profiles amongst returnees (121). Indeed, compelling evidence exists for the effectiveness of such workshops as a successful avenue to disseminate PA prescription strategies. However, in order to build and expand on EIMC's success, it is crucial that future workshops be informed by evidence. Accordingly, the aforementioned TDF may be used to identify more specific barriers and facilitators that physicians may face with PA prescription, such that interventions may be designed with the intent of targeting these barriers, making the intervention workshops more efficacious (111, 122, 123). Nonetheless, these workshops should (1) emphasize the importance of the cultural sensitivity in PA prescription for SACs and (2) recognize the sociocultural context of SACs' CVD risk; knowledge of these sociocultural variations may help to facilitate the delivery of PA programs that satisfy the cultural preferences of SACs, and ultimately enhance uptake and utilization (83), and also improve quality of care (49).

## Future directions

By 2020, The Heart and Stroke Foundation aspires for a 10% improvement in heart health of all Canadians (124). This goal can only be achieved by identifying high risk groups within a population, determining barriers and facilitators to preventative behavior like PA, and implementing efficacious interventions to address these barriers and augment facilitators. Undoubtedly, the prevalence CVD in SACs is amongst the highest in Canada (12, 13, 15, 16). They also suffer from a full spectrum of CVD risk factors, ranging from modifiable hypertension and diabetes (3, 13, 21) to non-modifiable adverse genetic profiles (125, 126). They also have unique barriers to preventative and curative PA habits, which include an unfavorable illness perception (44–46), previous cultural norms (37, 51–53, 81), substantial immigration-related matters (55, 60, 61), gender- and religion-related discrepancies (31, 73, 74, 80), a negative PA environment (79, 81), and a large chronic diseases burden (3, 6, 12, 13, 15, 21, 22), leading to one of the lowest PA participation rates in Canada (3, 6, 35).

PA prescription is a cost-effective intervention that could save the healthcare system significant expenditure if widely adopted, ameliorating the healthcare burden of preventable chronic disease, such as CVD. Bounajm et al. suggest that increasing PA levels in sedentary individuals with chronic disease can save the Canadian healthcare system an estimated 2.1 billion dollars in healthcare associated costs, such as hospitalization and diagnostics (127). Educating primary care physicians on the importance of culturally-sensitive PA prescription is an important step (8–10), with previous research describing methods to increase physician-prescribed PA (118, 120). Another critical aspect is addressing SACs' barriers to PA. Significant evidence illustrates the effectiveness of using behavior frameworks in identifying barriers and opportunities in PA participation and addressing them in an evidence-based manner (111–114), with many studies implementing PA programs to SACs in a culturally-sensitive manner reporting noteworthy success (28–31, 33).

## Conclusion

There have been significant strides in understanding the prevalence, impact, and nuances of CVD amongst SACs. While there are ever-increasing amounts of data on the benefits of PA in this context, there continues to be multi-factorial impediment to actualization of health improvement. This review highlights the importance of ongoing fundamental translational work in bringing a multi-factorial accounting of participation barriers, ranging from the biological to anthropological, toward enabling optimal clinician armament and improved patient care.

## Author contributions

TT conceptualized and initiated the mini-review, was involved in analysis, interpretation of data, and drafting of the manuscript. MI drafted the manuscript, wrote Barriers to Physical Activity in SACs section, and was involved in analysis and interpretation of data. SD and KD provided feedback and guidance throughout the completion of this manuscript. SK wrote Barriers to Physical Activity in SACs section. TT and SS wrote Clinician Knowledge and Physical Activity Prescription section. PG-R wrote the Conclusion section and provided guidance and feedback. NS wrote CVD Risk Factor Profile in SAC section. All authors were involved in critical revision of the manuscript for important intellectual content. The authors have no conflicts of interests to declare.

### Conflict of interest statement

The authors declare that the research was conducted in the absence of any commercial or financial relationships that could be construed as a potential conflict of interest.

## Abbreviations

AHP: allied health professional
ApoA1: apolipoprotein A1
ApoB: apolipoprotein B
BMI: body-mass index
CAD: coronary artery disease
CSEP: Canadian Society for Exercise Physiology
CVD: cardiovascular disease
EIMC: Exercise is Medicine Canada
HDL: high density lipoprotein
LDL: low density lipoprotein
MI: myocardial infarction
NAFLD: non-alcoholic fatty liver disease
PA: physical activity
SACs: South Asian Canadians
TDM: theoretical domains framework
T2DM: type 2 diabetes mellitus
VAT: visceral adipose tissue
WC: waist circumference
WHO: World Health Organization
US: United States
UK: United Kingdom.
